# DynamoVis 1.0: an exploratory data visualization software for mapping movement in relation to internal and external factors

**DOI:** 10.1186/s40462-021-00291-5

**Published:** 2021-11-04

**Authors:** Somayeh Dodge, Mert Toka, Crystal J. Bae

**Affiliations:** 1 Department of Geography, University of California, Santa Barbara, Santa Barbara, CA USA; 2 Media Arts and Technology Program, University of California, Santa Barbara, Santa Barbara, CA USA

**Keywords:** Animal tracking, Exploratory data analysis, Geographic visualization, Internal and external factors, Animation, Movement ecology

## Abstract

**Background:**

This paper introduces DynamoVis version 1.0, an open-source software developed to design, record and export custom animations and multivariate visualizations from movement data, enabling visual exploration and communication of patterns capturing the associations between animals’ movement and its affecting internal and external factors. Proper representation of these dependencies grounded on cartographic principles and intuitive visual forms can facilitate scientific discovery, decision-making, collaborations, and foster understanding of movement.

**Results:**

DynamoVis offers a visualization platform that is accessible and easily usable for scientists and general public without a need for prior experience with data visualization or programming. The intuitive design focuses on a simple interface to apply cartographic techniques, giving ecologists of all backgrounds the power to visualize and communicate complex movement patterns.

**Conclusions:**

DynamoVis 1.0 offers a flexible platform to quickly and easily visualize and animate animal tracks to uncover hidden patterns captured in the data, and explore the effects of internal and external factors on their movement path choices and motion capacities. Hence, DynamoVis can be used as a powerful communicative and hypothesis generation tool for scientific discovery and decision-making through visual reasoning. The visual products can be used as a research and pedagogical tool in movement ecology.

## Background

Developments in animal tracking and remote sensing techniques have created unique methodological opportunities to study animal movement patterns in relation to environmental and behavioral context information. Visualization tools have never been as widely available as they are today, creating a foundation for enormous strides in fields such as geography, information visualization, ecology, and biology. New multimodal sensors and data collection methods paired with powerful developments in spatial analytics and mapping tools have given scientists the power to study animals’ movement behavior and understand how they adapt given environmental variability [1]. These opportunities include combining new and increasingly abundant multifaceted data sets of animal movement tracking at high spatial and temporal granularity that are enriched with contextual information on the physiological state, motion and navigation capacities of the individuals, as well as the geography and the surrounding environment impacting their movement [2]. These new forms of data such as environmental data obtained from satellites and in situ sensors, as well as behavioral information recorded through ancillary bio-loggers and modern tracking sensors equipped with accelerometers, magnetometers, thermometers, etc. have been groundbreaking for understanding how changes to the internal state and external environmental context impact the movement strategies and behavior of animals [3–5].

Recent advancements in the field of movement ecology have demonstrated the importance of incorporating the contextual information in studying short-range movement and migration strategies [6–8], the interaction between individuals [9], the impact of human interference on animals’ activity and resource use [10], and more [11, 12]. Our ability to explore these patterns provides scientists with a more accurate understanding of the spatial and temporal influences on animal movement, which can support more impactful restoration and preservation techniques.

Visualization can play a significant role in ecological studies and conservation planning. It can enable researchers and decision-makers to explore complex spatial and temporal relationships between movement and its context, uncover hidden patterns, and convey information captured in the data. It can be used as a powerful tool to support hypothesis generation and confirmation through human visual reasoning and to facilitate communication and discussion in interdisciplinary research [1]. It can also be used as a decision-support tool to inform fieldwork and facilitate collaborations between experts and professionals from diverse backgrounds [13, 14]. Geographic visualization (in short, geovisualization) is a relatively new science with a root in cartography and mapping, facilitated by advances in computing, graphic displays, and extensive availability of spatiotemporal data [15, 16]. Many researchers spend entire careers developing and evaluating cartographic techniques and geovisualization approaches suitable for various kinds of spatiotemporal data. While incredibly useful, the geovisualization of movement data was previously challenging to conduct, as it required specialized programming or software experience as well as an understanding of the complex structure of spatiotemporal data and visualization techniques.

Today, there are many tools available designed for skilled professionals and researchers to visualize and map movement data. Examples include commercial Geographic Information Systems (GIS) (e.g., ArcGIS) or specialized open-source packages for movement data analysis in R (e.g., moveVis [17]) or in Python (e.g., movingPandas [18]). However, these packages either offer limited flexibility for design customization by the user to alter visualization forms and formats or require advanced programming and analytical skills and knowledge of data visualization. To respond to this gap, we introduce DynamoVis, as an alternative visualization tool that requires no knowledge of data visualization, coding, or specific programming languages. Relying on scientific and tested cartographic methods, DynamoVis allows users to forgo the rigor of developing necessary visualization techniques that take years of practice, and to quickly and easily create interactive static and dynamic visual representations of animal movement patterns as they correspond with environmental and behavioral factors.

The main contribution of this paper is the development of the open-source software, DynamoVis version 1.0, to enable movement ecologists to visually explore their movement tracking data in novel ways through interactive multivariate and animated visualizations, and map the interplay between movement and its embedding context as it relate to the internal and external factors described in the movement ecology paradigm by Nathan et al. [2]. The software offers a flexible design that enables the user to quickly and easily create custom visualization forms by incorporating a variety of visual elements (points, lines, vectors, background maps, time handles) and visual variables (color, shape, size, texture, position). DynamoVis can be used as a powerful communicative and hypothesis generation/confirmation tool for scientific discovery, decision-making, and education. Users of any background can design and record intuitive animations of movement data and share them with collaborators and the public. The visual products can be used as a research and pedagogical tool in movement ecology for knowledge dissemination, and to engage with decision makers, funders, and the general public.

An earlier beta version of the DynamoVis software (version 0.4) was first presented in a workshop paper by Xavier and Dodge [19]. The software was made available through the open GNU General Public License v3 and deposited in the Data Repository for the University of Minnesota [20]. Usage statistics from the Data Repository for the University of Minnesota show approximately 1,500 public downloads of the DynamoVis software from June 2018 to March 2021. The early version of the software was promoted primarily through specialized user workshops and via movebank.org^1^, an open and free repository for animal tracking data hosted by the Max Planck Institute of Animal Behavior [21]. Since the release of the beta version, there has been consistent interest in its use for movement data exploration and visualization, and many requests for a new version with an improved functionality. Responding to user feedback, this newly available stable and executable version of the DynamoVis software (version 1.0) includes several improvements in the user interface, data input, visualization elements, and functionality, making it reliable and useful for a broader audience. Additionally, previous issues and reported errors are resolved, including software incompatibilities with newer Java libraries for cross-platform support, input issues with reading newer data sets collected in the year 2020 or later, and previous gaps with the projection of data for longitudes near the 180th meridian. The software performance is greatly improved in the DynamoVis 1.0, thanks to code optimizations throughout the system and upgrading dependencies to newer, more efficient versions of Processing and Java libraries.

In the following sections, we provide an overview of the underlying cartographic framework of DynamoVis and its key features. Afterwards, we provide technical details regarding software implementation and the main functionality. Finally, we discuss important applications of the tool by providing several use cases for movement data visualization and exploration.

**Fig. 1:**
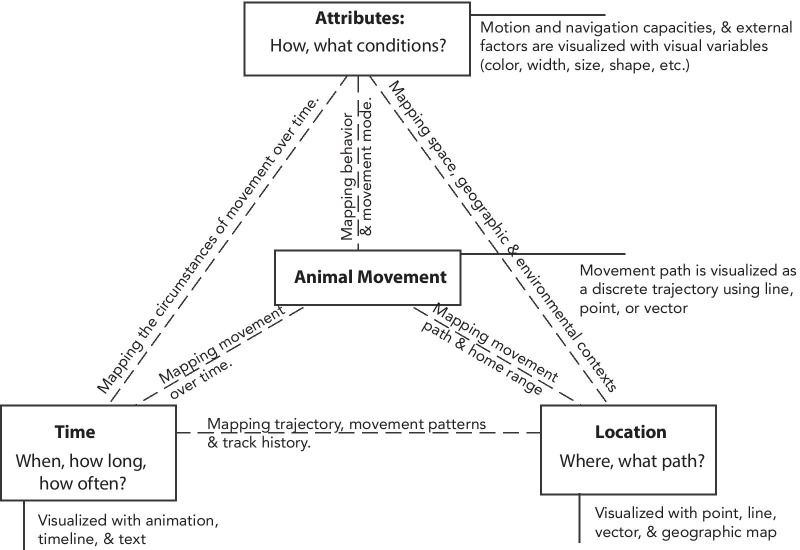
DynamoVis cartographic framework. The animal movement path is mapped using tracking point locations over time as points, lines, and vectors. Attributes related to motion and navigation capacities and external factors are mapped through the use of visual variables (size, shape, color, texture), and multivariate cartographic representations

## Cartographic framework of DynamoVis

DynamoVis is based on a cartographic framework introduced in Dodge and Noi [1]. Figure 1 provides a modified version of the framework tailored to the visualization of animal movement. The framework uses the pyramid model (object, location, attribute, and time) for the representation of geographic phenomena proposed in Mennis et al. [22]. Here, the geographic phenomenon (‘object’ or the subject of representation) is ‘Animal Movement’. Using this pyramid model, we map the main components of movement involved in the animal movement paradigm proposed in Nathan et al. [2] as follows:

**Location** describes the movement path as well as the geography within which the movement occurs. DynamoVis represents movement location using cartographic elements such as points, polylines, and vectors. The movement path is represented as a trajectory with a sequence of tracking points over time which can be connected via a trajectory polyline. The software also offers different choices for the underlying map to represent a frame of reference for the location and represent the geographic context surrounding the movement.

**Time** describes the beginning and end of the movement, as well as the frequency and duration of events and patterns, such as migration and stops. In DynamoVis, time is represented in two ways: as a linear visual timeline and the animation time. Both offer interactive features for users to navigate through time (i.e., move forth and back, speed up, slow down, start and stop animation) and visualize moving phenomena at different points in time. To identify the timing of the current tracking data in the visual display, DynamoVis adds the time information on the map as ‘text’.

**Attributes** describe variables relevant to the behavior (e.g., internal state) and the motion capacities of the focal individual as well as the characteristics of the movement path (e.g., speed, turn angle, path tortuosity, etc.). They also represent environmental variables and external factors (e.g., weather condition, vegetation, etc.) which may influence animal movement. In order to visualize these attributes, DynamoVis offers a range of multivariate visualization techniques through a combination of multiple visual variables, including color, line width, point size, vector direction, etc.

## DynamoVis key features

DynamoVis equips the users with a multitude of functions to create and customize unique multivariate representations of movement and context through a selection of different visual variables. The users can animate and explore their movement data, and export dynamic visualization outputs on demand using an interactive environment. First-time users can produce useful maps and animations within minutes of opening the software, creating an opportunity for movement data analysts to create complex visualizations in a simple, easy-to-use environment. DynamoVis also does not require knowledge of GIS for mapping generally and can be used as stand-alone software without a need for installation or integration of multiple packages and libraries. The point-and-click graphical user interface (GUI) of DynamoVis allows for sophisticated dynamic and interactive visualizations of movement with many customizable parameters and variables, which can be hard or almost impossible to achieve using existing GIS software packages. In addition to supporting open science and knowledge dissemination, the open-source features of DynamoVis make the software even more accessible to the scientific community and enables them to modify and advance its functionalities for specific applications. Below, we highlight other important technical features implemented in DynamoVis.

### Translating tracking data to movement animation

DynamoVis enables users to import any type of movement tracking data in comma-separated values (CSV) format. The data can include any duration of movement track for any number of individual animals and for any sampling intervals. The data can include tracking points of variable sampling intervals, and there is no software-imposed limitation in terms of the total number of tracking points or attributes, as we describe in the “Scalability and Performance” section. Further technical details about acceptable data specifications and input instructions are provided in “Input Data and Animation Configuration” section.

As an output, the software enables the users to capture static images of what represented on the screen and export videos of the created animations. The users are able to interact with the software and change the representation while the video recording is in progress, allowing for zooming, panning, or changing visualization parameters to create custom animations of different parts of the data. This is a unique function which can be leveraged for enhanced knowledge dissemination, presentation, and education purposes. Further technical details about the video recording function is provided in “Exporting Visualizations” section.

### Multivariate representations

DynamoVis provides the user with a range of visual variables to design and generate multivariate representations of movement paths of animals in the relation to any context factors that exist in the data. The user can customize how they visualize the tracking data using points, lines, directional vectors or a combination of all. Multiple attributes (e.g., including movement parameters, internal and external contextual factors) can be mapped over the movement tracks by selecting different visual variables (e.g., size, color, transparency, etc.) or a combination of them. For example, see Fig. 4 for a range of visual variables offered in DynamoVis. The section “Input Data and Animation Configuration” provides further detail on DynamoVis functions for creating and configuring multivariate representations.

### Interactive and custom animations

The most important feature offered by DynamoVis is to empower the users with interactive functions to design, record, and export custom animations for their movement data. The interactive, dynamic, and customizable aspects of DynamoVis provide an enhanced user experience for visual reasoning and exploratory data analysis. DynamoVis supports the highest level of human-map interaction, allowing the user to change the visual aesthetic of the representation on the fly, customize the visualized attributes through a range of visual variables, alter the map scale by zooming in and out, and explore different parts of the data by panning through the visualization. The user can change the speed of the animation and navigate the animation through a time bar to advance, rewind, or stop the presentation on demand. All changes made through the interactive display can also be recorded in the exported video on demand. Several examples of movement animations recorded using DynamoVis can be found on the Github page^2^.

The next section provides further technical details on DynamoVis functions to create and customize interactive animated representations.

**Fig. 2:**
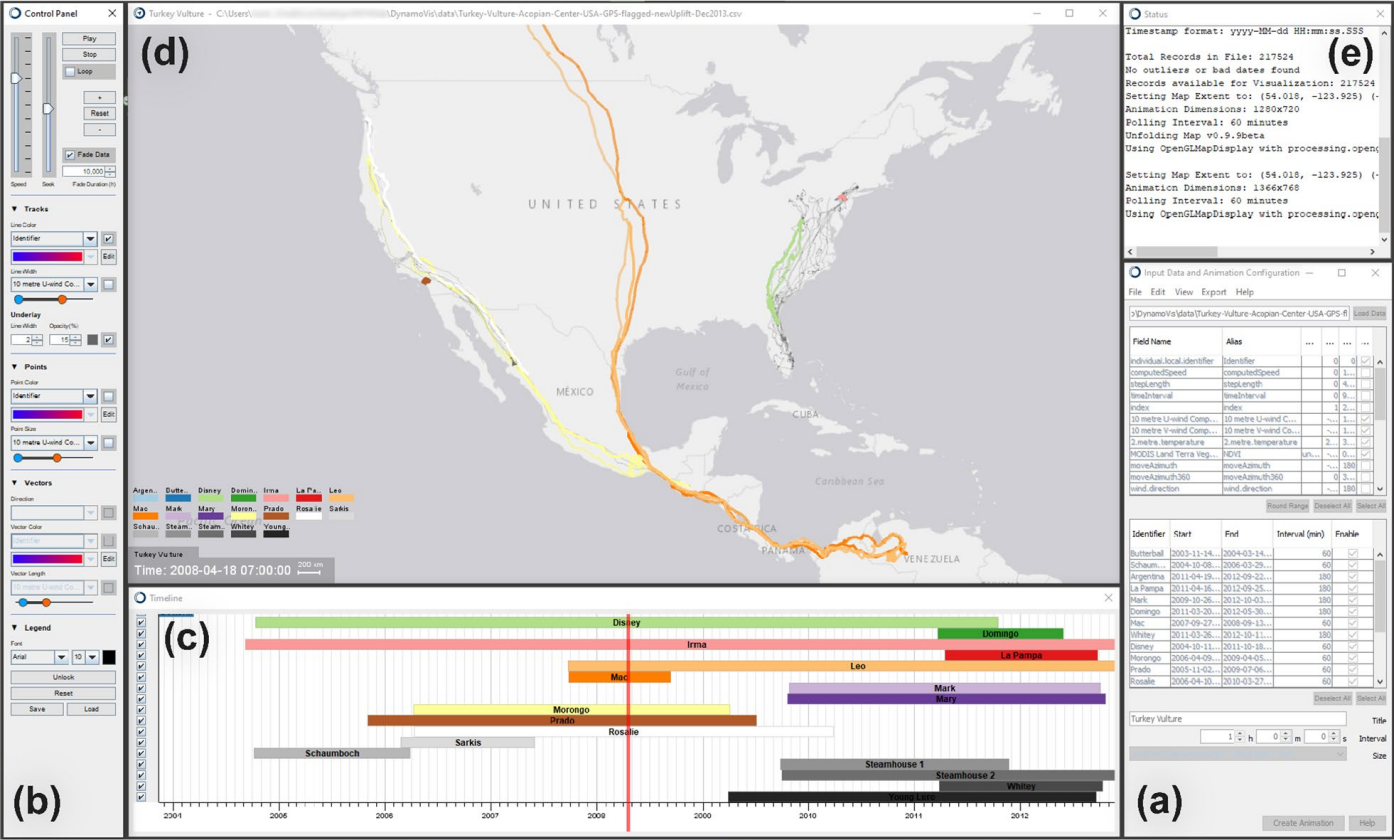
Components of DynamoVis 1.0: **a** *Input Data and Animation Configuration*, where the data is loaded, and required fields for visualization are selected, **b** *Control Panel* to set and manipulate various visualization elements, **c** *Timeline* to display individual animal tracks spatially, **d** the main *Animation* window to illustrate movement visualization and legend, and **e** the *Status* window which displays related messages about data loading and error monitoring. The *Animation* window visualizes seven turkey vulture tracks in North America on an Esri World Gray Canvas basemap. The data is accessible at 10.5441/001/1.46ft1k05 [7]

## Implementation and technical details

DynamoVis is developed as an open-source, cross-platform, standalone native desktop application using the object-oriented programming (OOP) paradigm. OOP is desirable since it allows for a modular implementation scheme and increased code reusability. These benefits are essential for the longevity of the software, as they lead to easy troubleshooting and maintenance of the codebase by various contributors in university labs or by the open-source community. We use Java, a popular OOP language, as a base for managing the data and GUI of various modules. GUI modules are created with Java Swing and Abstract Window Toolkit (AWT) components for their cross-platform reliability. The primary movement animation is implemented using Processing [23], an open-source Java library that abstracts tedious computer graphics commands away and provides an intuitive, user-friendly development environment. In the implementation of DynamoVis, Processing handles all graphical commands (trajectory shapes, annotations, and legend elements) on a geographic basemap. The open-source Unfolding Maps library [24] for Processing is employed for managing map functionalities. Unfolding Maps provides tile-based maps from various providers (e.g., Google Maps, Microsoft Aerial, and Esri World Terrain) as well as a set of core cartographic functionalities (zooming, panning, and projection mapping).

Figure 2 illustrates the main user interface and the different components of DynamoVis, as described in detail in the following sections. DynamoVis 1.0 features five main components in the form of multiple coordinated views: (a) *Input Data and Animation Configuration* to load the input data and select variables for visualization and animation, (b) *Control Panel* to modulate various visualization elements, (c) *Timeline* to display individual animal tracks spatially, (d) *Animation* to show the main spatiotemporal movement visualization and legend, and (e) *Status* to display message logs and monitor errors.

**Table 1:** Required fields in the input data set with their description, variable type, and value ranges

Field	Description	Variable type	Value range
Identifier	A unique identifier for each animal	Alphanumeric string	N/A
Longitude	The longitude coordinate of the tracking point	Floating-point	$[-180.0, 180.0]$
Latitude	The latitude coordinate of the racking point	Floating-point	$[-90.0, 90.0]$
Timestamp	Date and time of the tracking point	Datetime string	N/A

### Input data and animation configuration

Upon opening the DynamoVis software, the user is presented with the *Input Data and Configure Animation* window to load an input data set and configure the visualization parameters (Fig. 2a). Below, we elaborate on acceptable data formats and processing details, and describe the various functions of this view.

DynamoVis accepts any conventional tracking data (e.g., GPS or telemetry data) in the form of CSV files. The data may include any number of attribute fields, but it requires a minimum of four fields (Table 1): The *Identifier* field accepts any string or numeric variable and is expected to be unique for each tracked individual. DynamoVis uses this as the unique identifier for represented trajectories. *Longitude* and *Latitude* fields represent the geographic coordinates of the recorded points in the World Geodetic System 1984 (WGS84), and are expected to be floating-point numbers in the corresponding ranges in degree. The *Timestamp* field is formatted as a Java datetime value using a string. DynamoVis can interpret a wide range of datetime string formats (e.g., “yyyy-MM-dd HH:mm:ss”). However, if the string is formatted differently and the software is not able to parse the time field, and it prompts the user to enter their custom datetime string format using common forms such as “yyyy” for year, “MM” for month, and so on. This custom format is remembered for future use and only needs to be recorded once per data file.

**Fig. 3:**
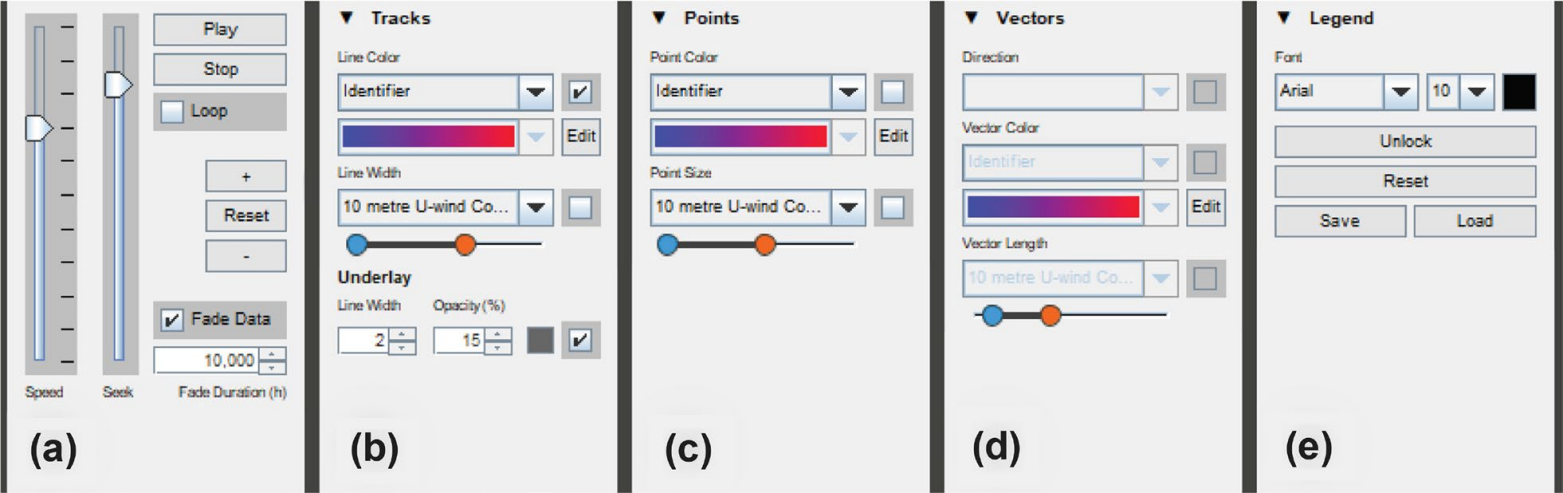
Components of the control panel in Fig.  2b, presented side-by-side. From left to right, it shows **a** the animation and map controls, **b** Track visualization controls and Underlay settings, **c** Point visualization controls, **d** Vector visualization controls, and **e** Legend options

By default, DynamoVis recognizes animal tracking in the format used in Movebank [21]. That is, for the pre-defined header titles of the required fields in the data header, the Movebank Attribute Dictionary^3^ is employed. As such, DynamoVis automatically looks for the default header names with “individual-local-identifier” for the *Identifier* field, “location-long” for *Longitude*, “location-lat” for *Latitude*, and “study-local-timestamp” for the *Timestamp* field. For data stored in other formats, DynamoVis prompts the user to select the columns in the data set corresponding to the required fields. Therefore, the user can use any movement data in CSV format by specifying which columns in the input data correspond with the required fields (as presented in Table 1), without altering the data set. In such cases, DynamoVis registers and remembers the introduced custom header names for the required fields after prompting the naming convention only once for each data file. The new custom headers are automatically used in future sessions for the same data file.

As two default optional fields, DynamoVis looks for “study-name” and “visible” fields which often are available in Movebank-generated data. If available, DynamoVis uses “study-name” field to assign a string as the title for the animation window and exported videos. Otherwise, the user can manually insert a name for the project. The “visible” field in Movebank data (i.e., a Boolean value representing erroneous ‘0’ or valid ‘1’ data points) can be used to determine if the particular row should be skipped for the visualization or not. This way, DynamoVis does not visualize outliers and erroneous tracking points in the data. Additionally, the user can use this field to manually annotate any tracking point that should not be considered in the visualization. In addition to skipping the whole row with “visible” field, the user also has the ability to discard irrelevant columns (attribute fields) that are not used for visualization (e.g., species name, battery information, sensor types, timezone, etc.) by adding the field names in the provided configuration file.

**Table 2:** Items in the summary of input data points; presented in the *Status* window upon loading the data

Summary item	Status of data points
Outliers with “visible” field manually marked as false or 0	Ignored
Corrupted coordinates	Ignored
Dates before 1800	Ignored
Dates between 1800 and 1980	Kept
Dates that are 20 years in the future of the current date	Kept
Dates that are further than 20 years in the future from the current date	Ignored
The total number of available data points for visualization	N/A

In addition to the required and optional fields described above, DynamoVis can read any number of additional attribute fields existing in the data as ancillary information. Examples include pre-computed movement parameters (e.g., speed, acceleration, direction), environmental variables (e.g., wind condition, temperature, perception, land-use, vegetation, snow cover), and behavioral information (e.g., migration season). For example, DynamoVis can read and visualize environmental variables that are annotated to the tracking data using the Movebank Env-DATA Track Annotation service [4]. Or, the user can compute and annotate any desired additional movement and environmental parameters, either automatically or manually, prior to loading the data to DynamoVis. These annotations can be formatted as integer or floating-point numbers for scalar and vector attributes. The current version of DynamoVis does not recognize categorical data and string variables (e.g., labels, modes) for attributes and these values needs to be encoded as integers for visualization purposes. For data sets that are annotated with the Env-DATA System, DynamoVis automatically assigns aliases and units to the ancillary data fields by referencing a repository of known environmental variables. A snapshot of this repository is placed under local configuration folder to read, filter, and annotate any data set locally without relying on networked solutions that could jeopardize data privacy and security.

Once the data is successfully loaded, the *Status* window (Fig. 2e) is updated with a running history of actions performed in the session. If the input data needs preprocessing before visualization, this window will print appropriate information about issues in the data set. DynamoVis 1.0 does not provide tools for filtering outliers or noise in the data; however, if any required fields are corrupt or empty, the software ignores the row and records it in a list of problematic data points. After loading the data, the *Status* window presents a summary of the recorded points to the user with the information in Table 2.

Upon loading the data, the *Input Data and Configure Animation* window shows all the field names from the input data along with their minimum and maximum values. If needed, the user can input aliases and units manually to each field to identify suitable labels for when field names appear in the *Legend*. There are options to round the ranges of the variables and enabling only the desired animals and data fields to include in the visualization. The bottom half of the window displays all individual tracks included in the data, denoted using the Identifier field. Checkboxes for each track can be used to enable or disable them in the output visualization. The user may also input an animation title, specify the appropriate time interval for the display, and select the screen size (or resolution, in pixels) for the animation window.

Finally, after setting up visualization parameters, the user creates an animation and the *Control Panel*, *Timeline*, and *Animation* windows appear in separate windows. By default, the map window is zoomed to the full extent of the data coverage, and is centered on the centroid location of the bounding box of the tracking points. All controls and fields on this configuration window are deactivated when an animation is running. The user can create a new animation to modify animation parameters.

### Control panel

The *Control Panel* window (Fig. 2b) allows the user to control the main visualization parameters and navigate the map and animation views. Figure 3 highlights distinct components of the panel side by side. The animation and map controls (Fig. 3a) control the playback, timeframe, playhead, and speed of the animation. This view also lets the user fine-tune the spatial zoom level of the map and reset the map view to encompass the bounding box of the entire animation.

**Fig. 4:**
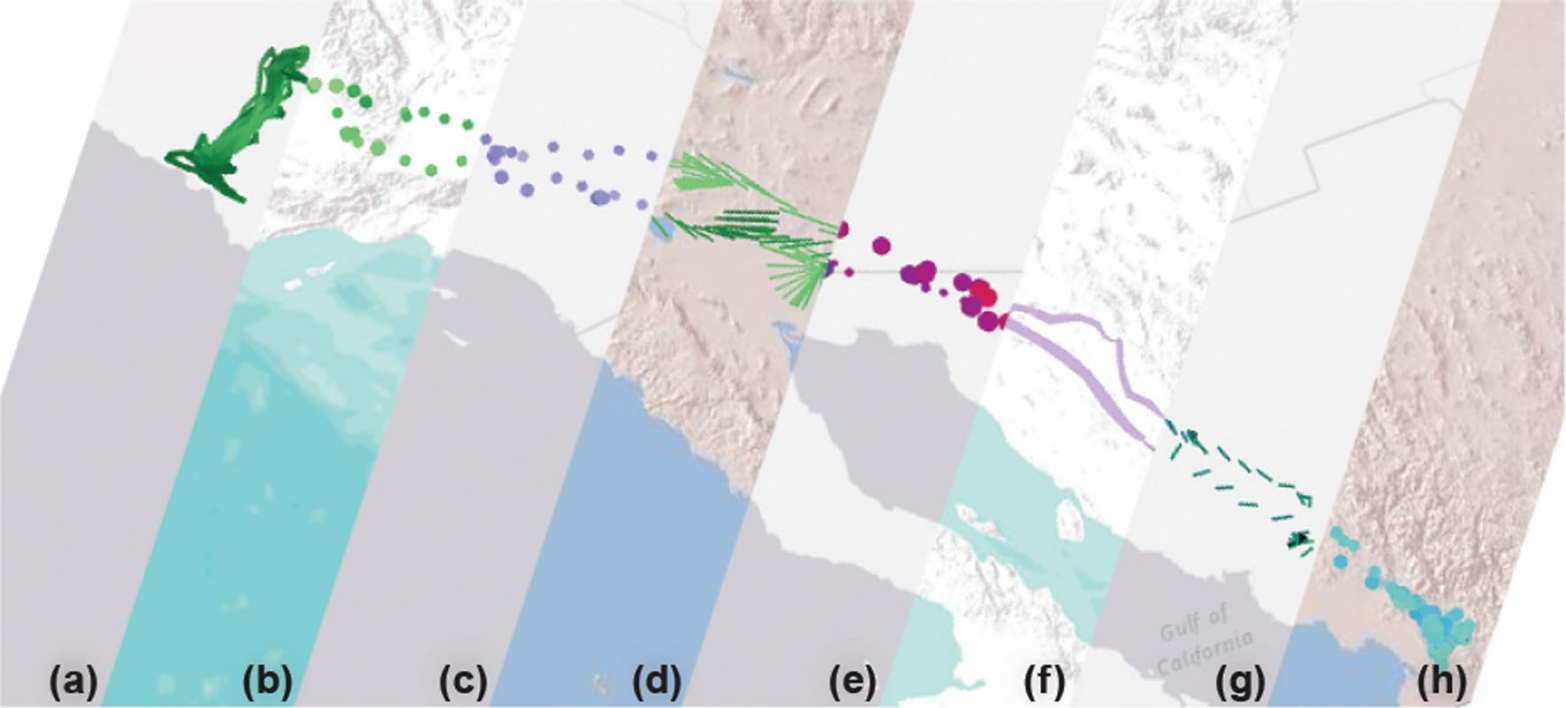
A collage of eight visualizations of the same track of a turkey vulture (Identifier: Sarkis) along the coast of Southern California and Mexico with different basemaps between February 2006 and June 2007. Visualization types; represented attributes; basemaps: **a** Tracks; line color and width represent *NDVI*; Esri World Gray Canvas, **b** Points; point color represents *Tail-Wind* and point size represents *NDVI*; Esri World Terrain, **c** Points; point color represents *Tail-Wind* and point size represents *NDVI*; Esri World Gray Canvas, **d** Vectors; vector direction represents *Wind Direction*, vector color represents *10 meter V-Wind* and vector length represents *2 meter Temperature*; Esri World Shaded Relief, **e** Points; point color represents *Wind Direction* and point size represents *Move Azimuth*; Esri World Gray Canvas, **f** Tracks; line color represents *unique color* for each animal and line width represents *Computed Speed*; Esri World Terrain, **g** Vectors; vector direction represents *Wind Direction*, vector color represents *Tail-Wind* and vector length represents *Computed Speed*; Esri World Gray Canvas, and **h** Points; point color represents *Tail-Wind* and point size represents *NDVI*; Esri World Shaded Relief

**Fig. 5:**
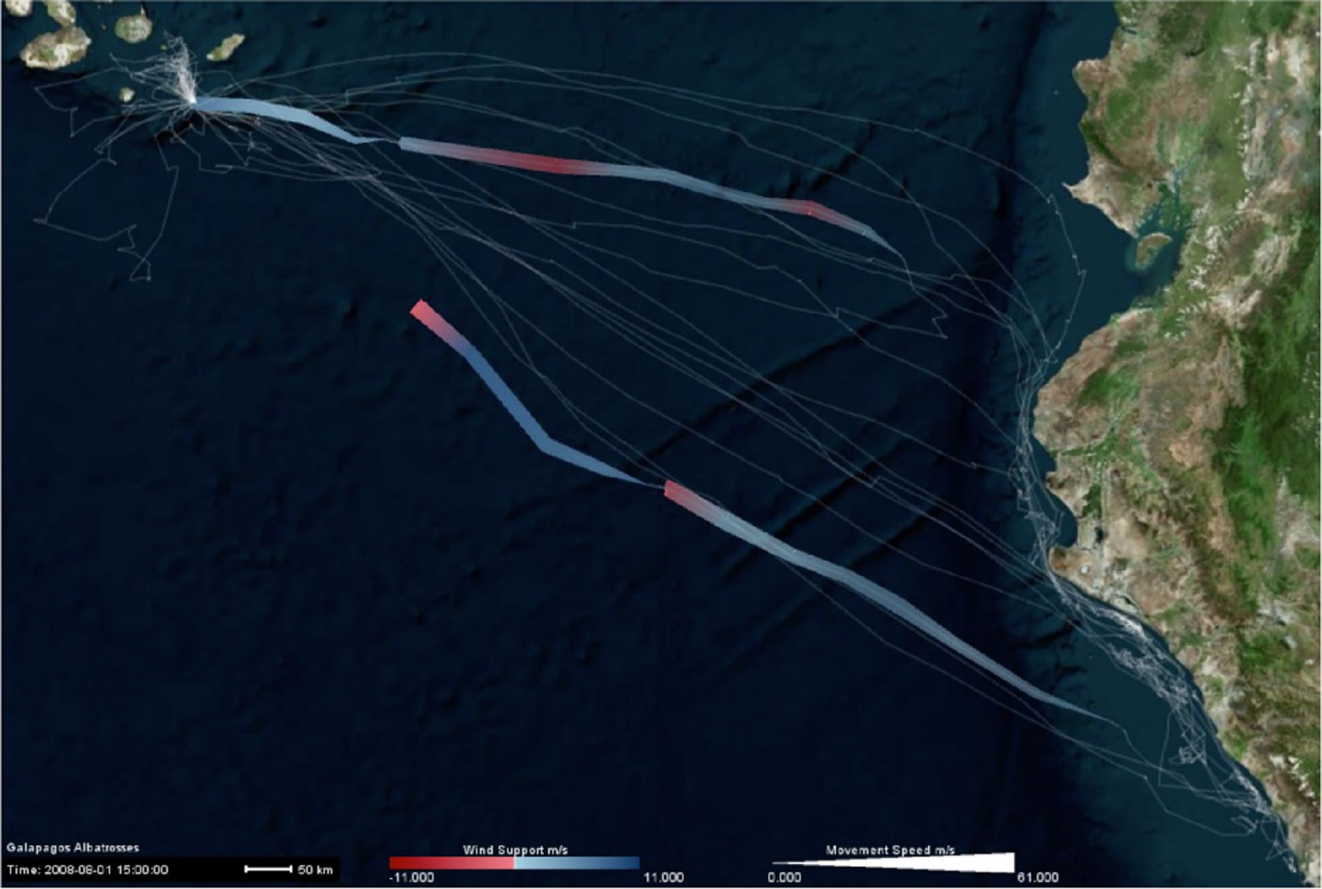
Fade data and underlay features. The map visualizes the movement of nine adult Galapagos Albatrosses (collected using 90 min GPS tracking between June and September 2008) [4] on the Microsoft Aerial basemap. Using the *Fade Data* feature, several hours of the recent trajectories are mapped saliently to highlight the association between movement speed (represented with track width) and wind support (tailwind is shown in blue hue and headwind is visualized in red). The previous history of the tracking data is represented using white narrow trajectory lines underlying the mapped tracks. The albatross tracking data are accessible at 10.5441/001/1.3hp3s250

Once the animated map is generated, all dropdown menus shown in Fig. 3b–d are populated with the data fields that have been selected during the initial configuration of the animation, including the unique animal identifier. There are three options for visual representations of tracking points: Line, point, and vector. To visualize different attributes (i.e., movement, internal or external variables selected from data fields) along the tracking points, multiple visual variables are offered: Line color, line width, point color, point size, vector direction, vector color, and vector length. As illustrated in Fig. 4, DynamoVis offers a wide range of multivariate visualizations of movement data through a combination of these visual variables. It is important to note that this figure merely present the range of visual variables that can be used for different attributes and the selection of basemaps, without necessarily conveying a narrative about the data. The design provides flexibility for the user to apply any combination of the visual elements and variables to map various attributes in the data along with the movement tracks. For example, the user can map the size or color of the respective visualization shapes (i.e., line, point, vector) to any selected attribute using the dropdown lists. As described later, Figs. 5, 7, 8, 9, 10 illustrate different applications of these visual elements for mapping movement and environmental variables along animal trajectories.

The color gradient dropdowns (shown in Fig. 3b, c) allow the selection or creation of new color ramps for mapping the attribute values to the colors. Note that selecting the *Identifier* property as the color attribute will always assign a unique color for the respective unique identifier and not a gradient ramp (see tracks shown in different colors in Fig. 2).

The double-slider bar with blue and orange ends scales the ranges of mapping attribute values, such as controlling the minimum and maximum thickness of the line in the *Tracks* panel. In the *Points* panel (Fig. 3c), it modulates the point size of the tracking points and, in the *Vectors* panel (Fig. 3d), the length of the vector that is drawn from each tracking point and its selected attribute. The *Vectors* panel can also assign another selected attribute to the direction of this vector. The checkboxes next to the dropdowns allow the user to freely enable/disable any number of combinations of these visualization options on demand.

In order to improve user cognition and reduce clutter in the visualization, DynamoVis offers the *Fade Data* feature, using which the user can fade the old data points in the track history and control the duration of track that should be visible prior to the current animation time. *Fade Duration* (in Fig. 3a) controls the duration (in hours) for which previous data points in the history of the track remain visible before gradually fading away, akin to specifying the temporal range for a moving window. That is, only the recent tracking points of a specified *Fade Duration* window are shown in the animation, and the rest of the tracks are made invisible.

Together with the *Underlay* feature (in Fig. 3b), *Fade Data* can guide the user’s attention to better navigate the animation by making recent data more salient while providing a map of the full track history in the background as a frame of reference. When enabled, the *Underlay* feature displays an overall history of the movement track up to the current point of the track display. This feature is helpful when used in conjunction with fading in animation controls when the user wants to see both the current tracking data and the overall trajectory traveled so far. It also provides a global overview map of the movement track and highlights the frequently visited locations at the end of the animation. The user can adjust and customize the visual appearance concerning the line weight, opacity, and color of underlay lines.

Figure 5 illustrates an example application of the *Fade Data* and *Underlay* features. Here, the tracking data of nine Galapagos Albatrosses (*Phoebastria irrorata*), collected from the period of June to September 2008 [4], are mapped. While the *Fade Data* feature only maps a window of few hours of recent tracking data on the current display, the *Underlay* feature visualizes the entire history of the data set as shadows of the trajectories using light white lines. The color, width, and transparency of the *Underlay* track is adjustable using the options shown in Fig. 3b.

In the output visualization, DynamoVis provides a flexible legend which can be customized and placed on the map by using the *Unlock Legend* feature in the *Legend* panel (Fig. 3e). The user may alter the position of the seven legend blocks displayed as an overlay on the *Animation* window: Title, line color, point color, vector color, line width, point size, and vector length. The user can select the text’s font and size as desired and store the custom legend layouts to reuse them for other animations. This allows for easy customization of the legend’s position and appearance on the animation, and the ability to create a consistent look across multiple animations.

### Timeline navigation bar

The *Timeline* window (Fig.  2c) visualizes the temporal relationship between all displayed movement tracks, labeled by their identifier. The red vertical line displays the current position (time) of the animation and can be manipulated through the *Control Panel* window. Additionally, within the *Timeline*, the user may use the checkboxes to enable or disable the visualization of specific animal identifiers on the fly. Clicking on the colored bar for a specific identifier highlights the track of the selected individual in the main visualization window, allowing for easy visual identification of the trajectory of individuals of interest.

### Animation

The *Animation* window (Fig. 2d) shows the main visualization in which the visual representation of the movement data is displayed and animated. Users can zoom in and out by large steps by using the mouse controls or in small steps using the *Control Panel* window. The map can be panned by dragging within the visualization window. The users can also reset the animation window to default extent using the *Control Panel*.

As previously discussed, the visualization of data attributes and movement tracks in the *Animation* window can be customized using different visual variables (e.g., lines, points, and vectors) via the *Control Panel* and *Timeline* windows. The animation reacts to the modifications in these panels immediately (see Fig. 4 for a collage of different visual variables). A legend item for each each visualized attribute is displayed in the *Animation* window along with general information about the data (i.e., animation title, current time, and map scale). The placement of these legend items can be adjusted using the *Control Panel* to prevent occlusion with other legend items and the points of interest in the animation.

To visualize the geographic context of the movement (landscape, land-use, topography, geographic extent, etc.), DynamoVis offers a range of basemaps (e.g., topographic, satellite imagery, road maps, and gray canvas maps) in the *Animation* window obtained from various web service providers such as Google, Esri, and Microsoft. Figure 4 highlights the variety of visualization types and basemaps that available from Esri web mapping services. DynamoVis currently supports the following list of basemaps to be used in animations: Esri NatGeo, Esri Ocean Basemap, Esri World Gray Canvas, Esri World Shaded Relief, Esri World Terrain, Esri World Topo, Google Maps, Google Maps Simple, Google Terrain, and Microsoft Aerial.

The animation window in DynamoVis 1.0 gracefully handles the data points and basemaps around 180th meridian. In the previous release of DynamoVis (0.4), map tiles were abruptly cut around data points with ±180 longitudes. DynamoVis 1.0 addresses this crucial issue by extending the underlying basemaps and correctly handling the projection of spatial points to prevent sudden jumps when crossing the 180th meridian.

DynamoVis 1.0 implements spatiotemporal movement visualization as a dynamic and interactive animated map. In order to make rendering highly efficient, the *Animation* window employs batch rendering, as opposed to sequential draw calls, to render data points and vectors. This optimization makes DynamoVis suitable for moderate hardware, and makes it particularly adept at visualizing larger movement data sets and those that are collected at higher recording rates or span longer time intervals.

**Fig. 6:**
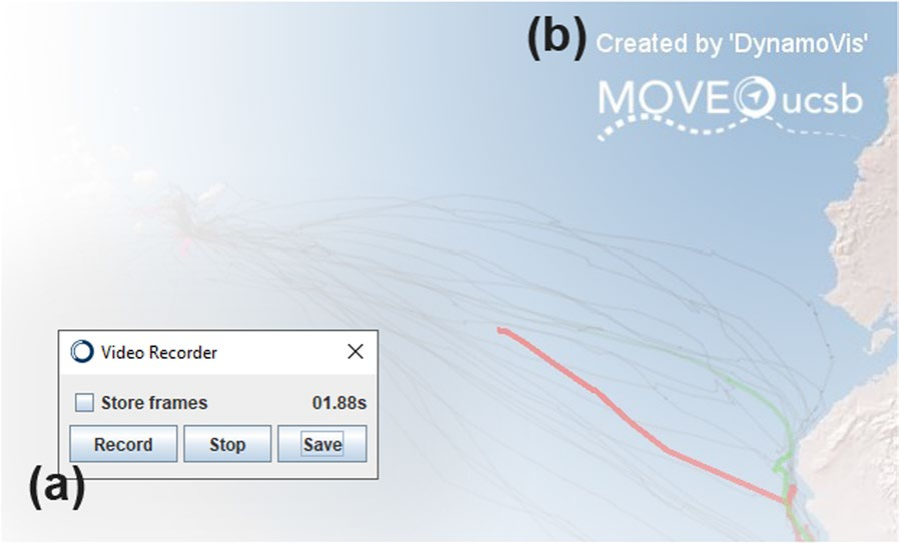
Exporting a recording of the animation. **a** The *Video Recorder* window that can be used on demand to record and save visualization outputs, and **b** watermark added in the upper-right corner of the recording

### Exporting visualizations

To record an animated map of movement data and export it to a video file, the user can use *Video Recorder* window accessible from the menu bar (Fig. 6a). The option to *Store frames* will add each recording frame as a separate image file in a new folder. Selecting *Record* will begin recording a video; the timer displays the current length of the recording in seconds. Once the software is recording, any change in the *Animation* window (e.g., zoom in/out, panning, changes in visual elements, and manual movement along the timeline) will be recorded in the final export. The user can pause the recording with *Stop* and resume it with *Record* as often as needed, such as for recording non-contiguous segments. Closing the *Video Recorder* window discards the current recording. Once finished recording the visualization as desired, selecting *Stop* and *Save* starts the encoding and export process. A progress window will show the status of the video encoding. The recorded video file will then be available with the animation title—specified previously in the *Input Data and Configure Animation* window—numbered by recording number in the /export folder of the DynamoVis installation location. A watermark is automatically added that displays the software information in the upper right-hand corner of the recording (Fig. 6b). If the *Store Frames* option was selected, each recording frame persists within the /export/temp folder.

### Scalability and performance

DynamoVis can accommodate large sizes of data to visualize movement trajectories. There is no limitation in the software for the total number of tracking points; however, the user may experience an upper-bound depending on the capacities of their computer’s random-access memory (RAM). We tested DynamoVis with the turkey vulture data set containing 217,525 tracking points and 22 additional fields for each point. This data set covers 19 migratory turkey vultures for a period of 112.5 months with a sampling interval of 1–3 h. Using this data set, the real-time memory requirement of DynamoVis 1.0 is just 1.5 GB.

Measured in frames-per-second (FPS), the visualization is considered real-time at around 24 FPS. In DynamoVis, the performance of the visualization and exported animation depends on the total number of tracking points visible at a given time. We tested this performance using the turkey vulture data set. With *Fade Data* toggle activated and *Fade Duration* set to the default value, we achieve real-time performance throughout the duration of the data set (112.5 months). However, when we disable the *Fade Data* toggle and draw all points that have occurred up to the seek position, the frame rate drops below real-time after the 40th month ($\sim 35\%$ of the timeline). For larger data sets, it is recommended to employ the *Fade Data* feature with a desired *Fade Duration* for better performance and smoother animations.

We tested DynamoVis with modern computers running Windows 10, Windows 11, and MacOS Catalina to ensure compatibility with multiple platforms. We note that it also works on Ubuntu, but we have not run exhaustive tests on this platform. The major specifications of the primary testing computer are as follows: Intel i9 CPU, NVidia RTX 3080 GPU, 64GB RAM running Windows 10.

## Results: use cases

The flexibility of DynamoVis allows for sophisticated visualizations of the relationship between movement and the influence of internal and external factors, which can help to portray how these factors may impact movement choices of animals. In this section, we demonstrate how DynamoVis can facilitate producing informative visualizations for data exploration with rich environmental and geographic context information.

### Data sets

We highlight various features and visualization outcomes using two data sets mentioned earlier: (a) migratory turkey vultures described in [7], and (b) foraging flights of Galapagos Albatrosses described in [4]. Both data sets are publicly available via Movebank (see “Data accessibility” section). The data sets are annotated using the Env-DATA system [4] with a set of environmental variables such as tail-wind, cross-wind, and thermal uplift^4^, Normalized Difference Vegetation Index (NDVI) from MODIS^5^, Ocean net primary productivity (NPP)^6^, and computed movement speed. The process of data collection, preparation, and annotation is detailed in Dodge et al. [7] for the turkey vulture data, and Dodge et al. [4] for the albatross data.

DynamoVis 1.0, the accompanying visualization examples, and the data for the experiments described in this section are accessible via the software’s GitHub page^7^. For better perception of the dynamic movement patterns presented here, the animated version of all the visualizations are also provided via the project’s Github page.

### Visualizing flight strategies

Previous studies [4, 7, 25] revealed existing dependencies between flight path choices of foraging albatrosses and migratory turkey vultures and meteorological conditions. Examining these associations can help scientists see a more complete picture of animal’s movement in conjunction with the external environmental context. Using DynamoVis, we illustrate how these birds utilize the meteorological conditions to facilitate their flights. We demonstrate the associations between movement speed, as an internal variable, and the external factors such as wind support for albatrosses, and thermal uplift for turkey vultures.

**Fig. 7:**
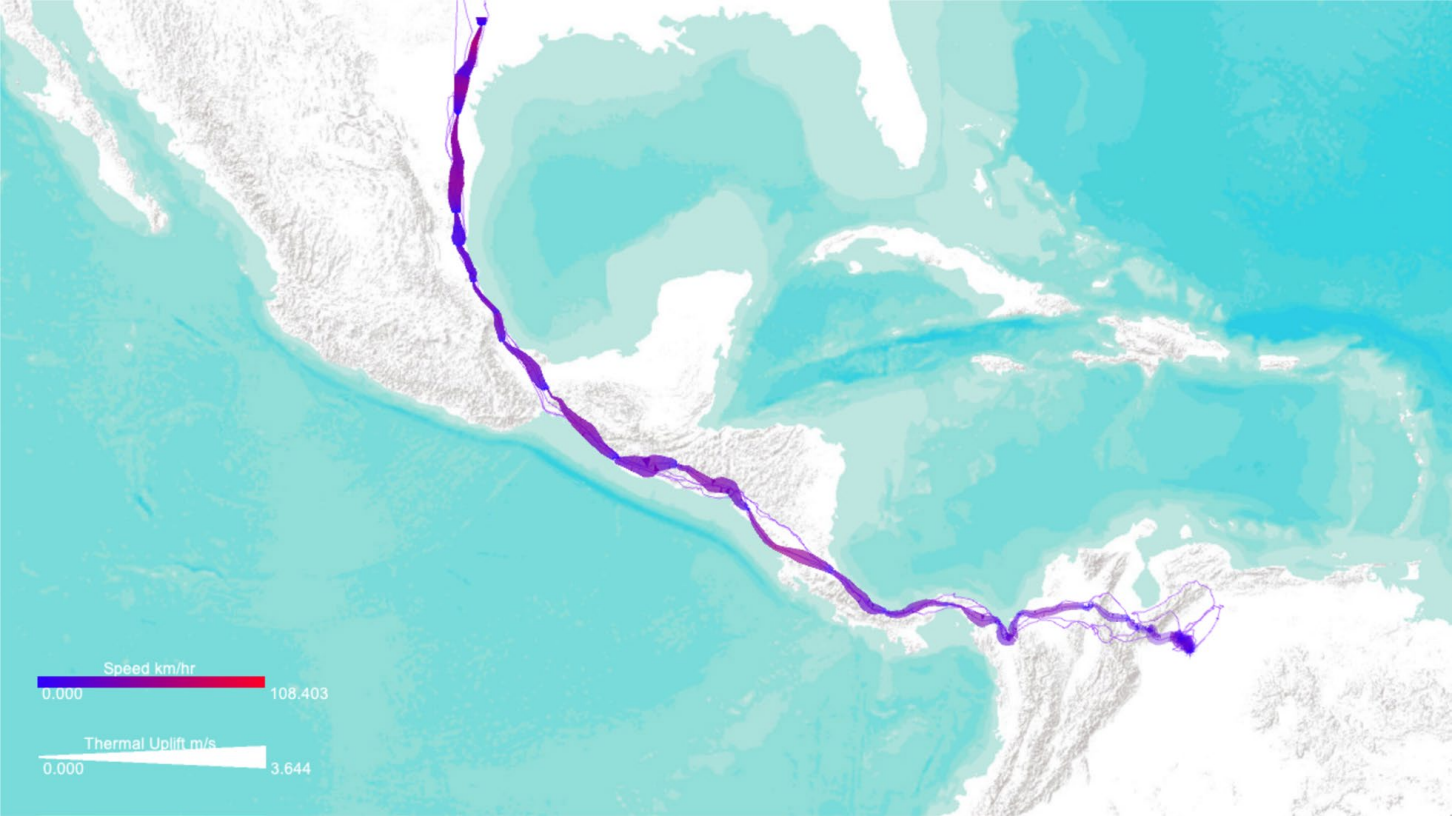
A turkey vulture track (Identifier: Leo) displayed on Esri World Terrain basemap, illustrating the relationship between the turkey vulture’s movement speed and thermal uplift. The visualization shows that the bird moves faster when thermal uplift is higher (shown with a thicker red line). The narrow underlay shadow shows the prior movement history of the turkey vulture (i.e., between September 2007 and October 2009), suggesting commonly-traveled routes. The data is accessible through 10.5441/001/1.46ft1k05 [7]

Turkey vultures are obligate soaring migrants, which means they rely heavily on atmospheric conditions for migration. As turkey vultures often do, traveling over long distances limits time for rest and feeding, highlighting the importance of low-cost soaring. Studying movement patterns helps to reveal how turkey vultures rely on thermal corridors to reduce the energy cost of flying [7, 25, 26], whereas other species may be more dependent on other environmental conditions such as prevailing wind speeds [27]. For example, Galapagos Albatrosses rely on wind to optimize their return flights to Galapagos after foraging along the Peruvian coast [4].

Using GPS tracking data of one turkey vulture (identified as Leo) during its Spring migration (from South America to Canada), Fig. 7 uses *Line Width* to represent thermal uplift, with higher uplift values shown with thicker trajectory lines, and *Line Color* to represent movement speed, with red color for higher speeds and purple for slower speeds. Therefore the software allows us to visualize when and where the animal moves rapidly or slowly, and how the bird’s speed relates to thermal uplift. Using Underlay, the history of the turkey vulture’s movement over the entire recorded time period is shown as well. The figure shows the movement of the turkey vulture as it travels from Venezuela (in the lower right corner of the image) through northern Colombia and continuing northwest along the Central American isthmus as it continues its migration. Lower-speed portions of the movement track, shown in blue, appear to correspond with lower amounts of thermal uplift, shown in the figure by the narrow portions of the track. The bird used a higher thermal uplift to speed up in the southern part of Guatemala (thicker red lines), while it moved relatively slower prior to that with low thermal uplift. This bird’s past movements are shown with the thin purple underlay line in Fig. 7. Clearly, this turkey vulture travels more regular migration routes across Central America, but demonstrates varied patterns over Colombia and Venezuela.

**Fig. 8:**
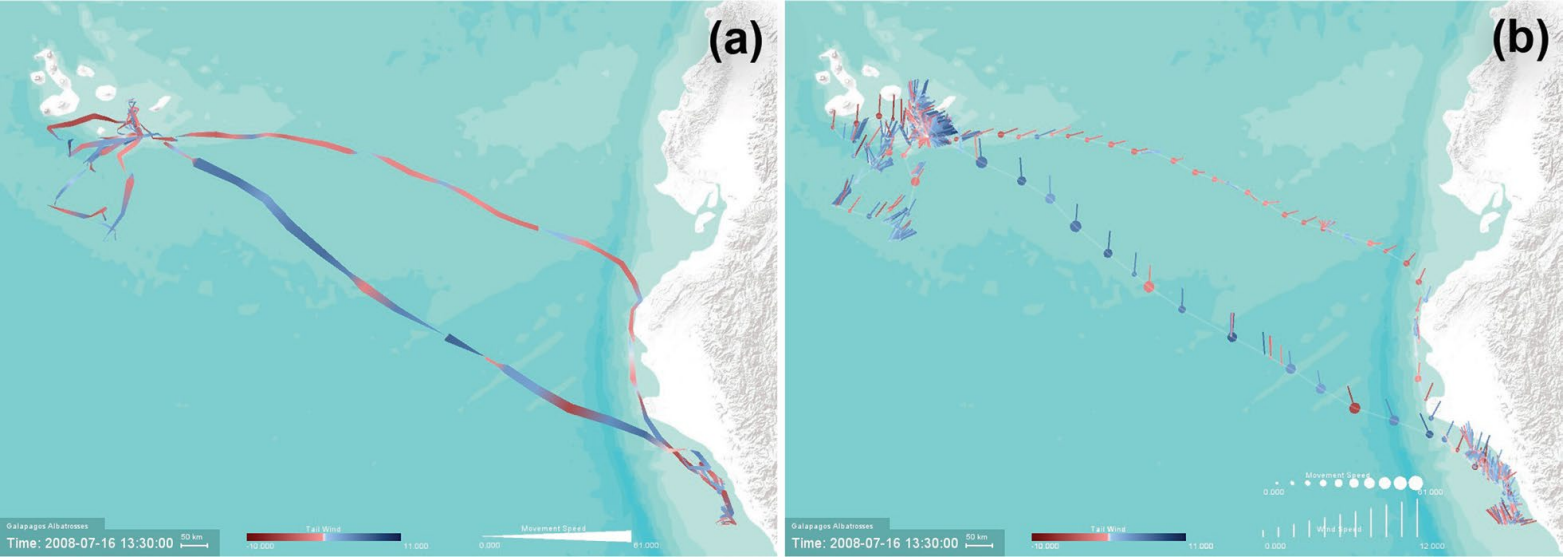
A Galapagos albatross track (Identifier: 4264-84830852) displayed on Esri World Terrain basemap between June and July 2008, illustrating the relationship between the movement speed and wind support. **a** The visualization highlights the use of higher tailwind support (shown in blue color) for faster outbound flights (shown with thicker lines). **b** The map highlights the actual wind direction using vectors of variable size (representing the wind speed) as it relates to the movement speed (represented as point size) along the path

Using GPS tracking data of an albatross, tracked during its breeding season between 31 May 2008 and 19 July 2008 with a temporal resolution of 90 min, Fig. 8 illustrates two different forms of visualizations highlighting the albatross’s use of wind assistance, in particular, during the return foraging flight back to Galapagos. Dodge et al. [4] demonstrated that Galapagos Albatrosses are often challenged by wind during their outbound foraging flights to the coast of Peru, while they wait for a favorable wind to fly faster back to their nest. Figure 8a uses *Line Color* to represent tailwind support (blue) and headwind (red), *Line Width* to visualize variations in movement speed. It highlights the albatross’s strenuous flight (thinner red lines representing slower movement with lower tailwind assistance) during the outbound flight, and the faster wind-assisted flight (thicker blue lines representing higher movement speed and tailwind) along a direct path back to Galapagos. Figure 8b uses *Vector Direction, Length, and Color* to better highlight the actual direction of the wind using vectors of variable sizes (representing the amount of tailwind support) as it relates to movement speed (visualized by *Point Size*) along the albatross flight path.

**Fig. 9:**
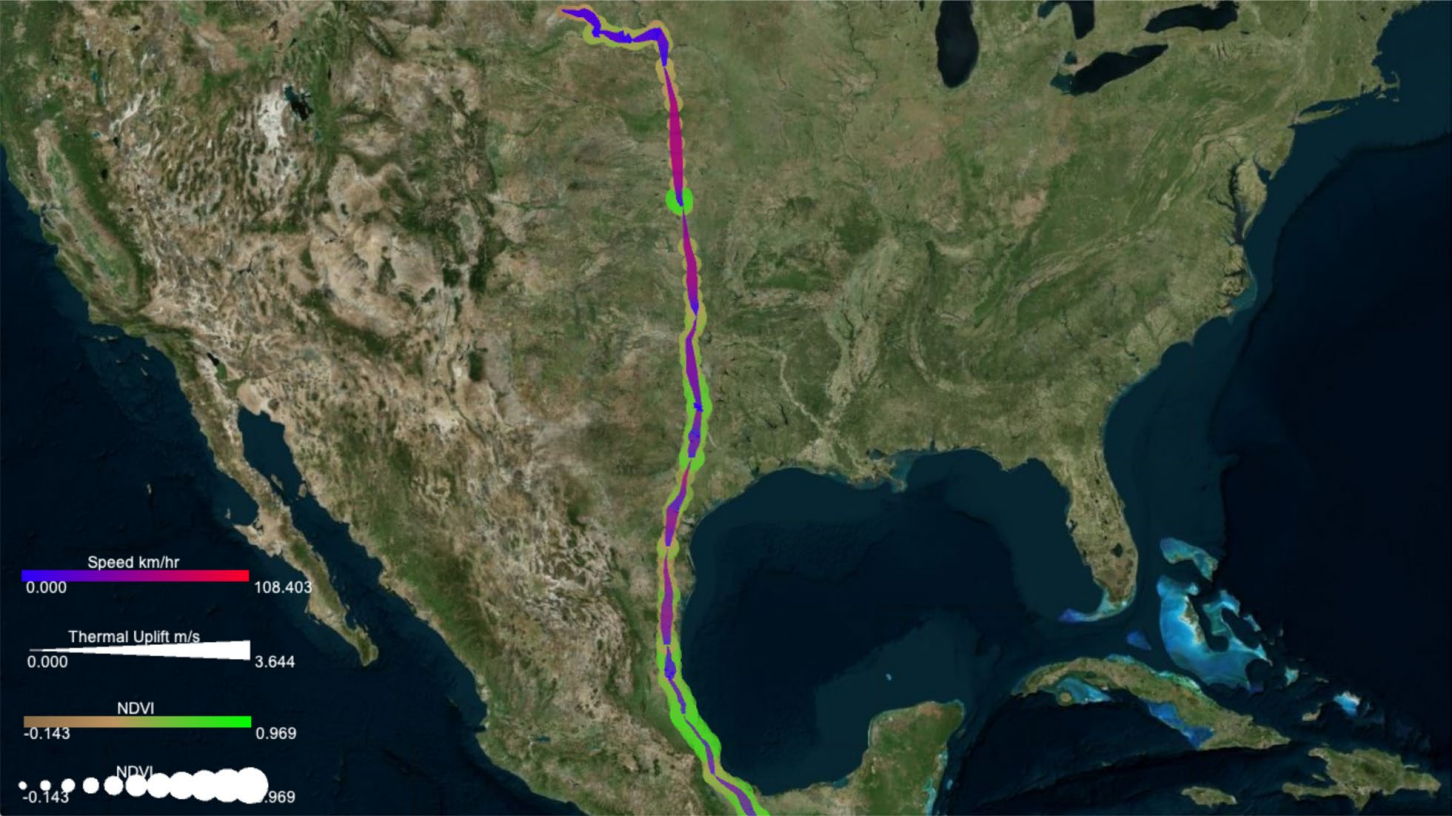
A turkey vulture track (Identifier: Leo) displayed on Microsoft Aerial basemap between March and May 2009. The visualization highlights the relationship between NDVI and turkey vulture movement speed. The turkey vulture appears to move slowly or pause in high-NDVI areas, shown with bright green circles. The bird’s movement speed is aided by thermal uplift

### Visualizing foraging strategies

So far we demonstrated what we know from the existing analytical studies [4, 6, 7, 25] about these birds’ strategies in choosing environmental conditions that optimize their flight paths in terms of spending less energy to reach a higher movement speed. Next, we explore possible environmental variables that may impact the forging strategies of these birds.

Figure 9 visualizes an additional environmental variable—in this case NDVI to represent healthier vegetation—using *Point Size* and *Point Color* along the same turkey vulture’s migration path. The track color represents movement speed, the track width represents thermal uplift, and the point size and color visualize the NDVI measurement at the tracking point. Areas along the track with a higher vegetation index are shown in larger, bright green circles, and lower NDVI values are shown in smaller, tan circles. With this information in mind, it becomes apparent that turkey vultures are habitually traveling to places with healthier vegetation with higher NDVI values, quickly passing through more sparsely vegetated places. That is, in areas of lower vegetation, we interpret that the bird moves faster with fewer foraging stops, as shown by brighter and redder lines over tan circles. In contrast, the bird makes more foraging stops and shows slower movement on average in areas of higher vegetation density, seen by the lines which are more blue over larger and greener circles. We also observe that the bird’s movement speed appears to be aided by thermal uplift, because for the most part the track is brighter and redder in places where it is thicker.

**Fig. 10:**
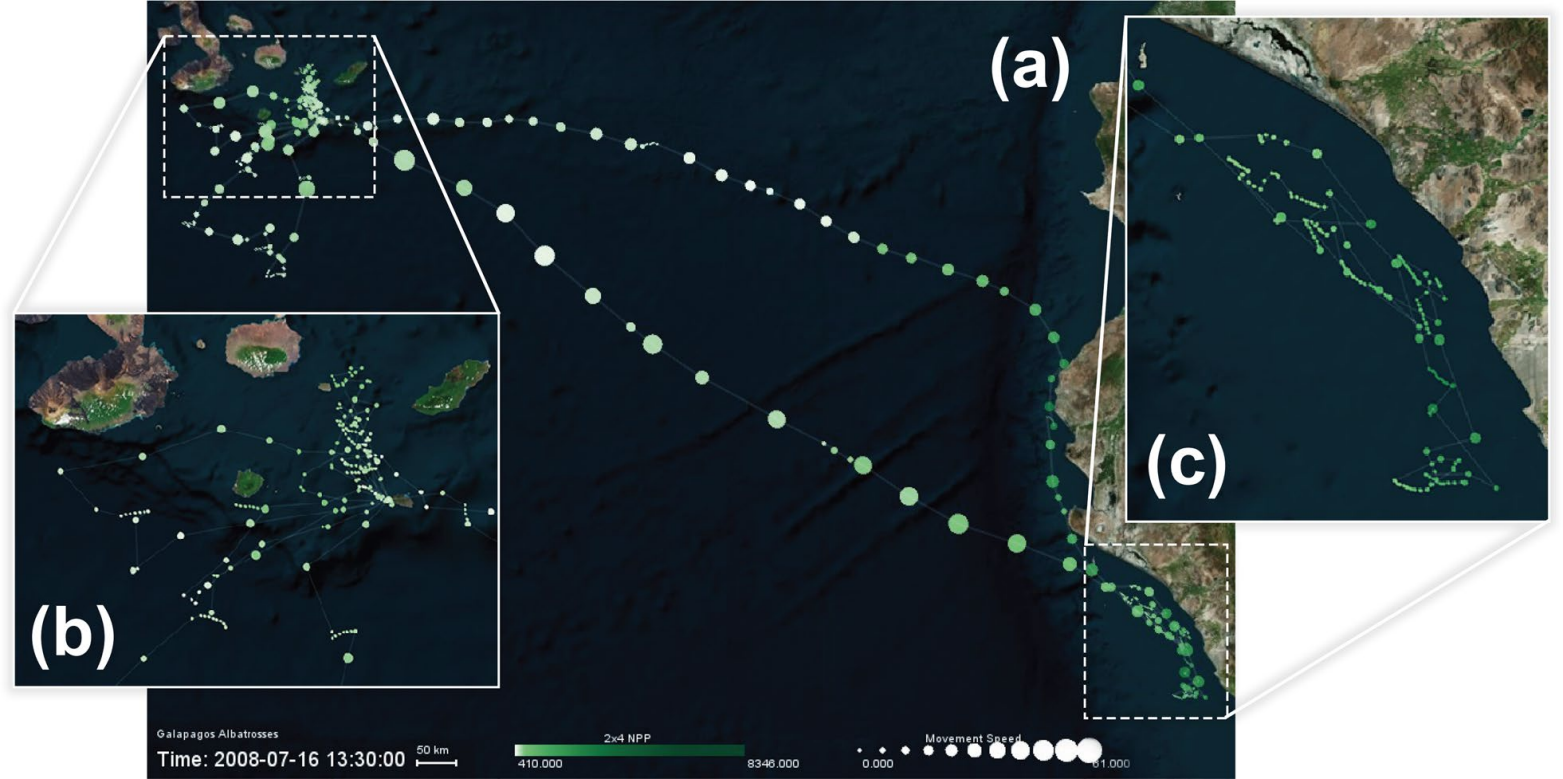
An albatross track (Identifier: 4264-84830852) displayed on the Microsoft Aerial basemap between June and July 2008. The visualization highlights the relationship between net primary production (NPP) of the ocean and albatross movement speed. The albatross appears to move slowly (smaller point size) or make frequent stops along the coast of Peru to forage in high NPP areas (shown in darker green colors). The bird appears to fly faster (larger point size) during the flights over the ocean and around the Galapagos where ocean NPP is lower (lighter green colors). **a** The overall foraging route of the bird, **b** close-up of the bird’s activity around Galapagos Islands, and **c** close-up of the bird’s activity at the coast of Peru

Figure 10a visualizes the Ocean NPP (represented bi *Point Color*) as an indicator of food availability in the ocean [4] contrasted against movement speed (represented by *Point Size*) along the flight path of the same albatross. As the figure highlights, Ocean NPP is the highest (darker green colors) at tracking points recorded along the coast of Peru. In these areas, the bird shows a slower foraging behavior as shown by smaller tracking points that are recorded close to each other along the track, indicating that the bird did not move much in those areas and made frequent stops (see Fig. 10c). In contrast, along the flight paths between the coast and Galapagos as well as in the areas around the Galapagos (see Fig. 10a, b), the bird often moves faster (shown with larger points) at locations with lower Ocean NPP (lighter green colors). The bird also seems to be displaying frequent stop and search behavior (i.e. closely recorded tracking points demonstrating very low speeds) around the nest location in the Galapagos.

## Discussion

The above visualizations demonstrate how DynamoVis can be used to uncover hidden patterns in the data, and hence, it can be used as a powerful communicative and hypothesis generation tool for knowledge construction, communication, and scientific discoveries. The outcomes can also facilitate confirming and communicating known patterns through visual reasoning. However, we should note that, although the experiments demonstrated DynamoVis flexibility for multivariate visualization of various attributes at the same time through a range of different visual variables and map elements, ﻿it is important that the analyst uses their expert judgment to decide which variables may relate and work well together to avoid unnecessary visual clutter and irrelevant views, as well as misleading interpretations. DynamoVis is mainly intended to enhance data exploration experience and human performance in knowledge discovery and hypothesis development through visual reasoning. As such, observations and interpretations from the visualizations are not intended to be conclusive, or to replace the need for quantitative data analysis. However, they can support data analysis and facilitate communication in the research process by streamlining data visualization and exploration and helping researchers to select relevant attributes prior to formal data analysis.

DynamoVis can also help to facilitate research discussions in interdisciplinary and collaborative work and communicate results by illustrating significant findings from the analysis. For example in our collaborations with the experts who tried DynamoVis products, they described it as a “*powerful hypothesis generation and knowledge communication tool*” that helps “*ideas emerge by exploring [tiger tracking] data*” (Prof. James L.D. Smith, Department of Fisheries, Wildlife and Conservation Biology, University of Minnesota). Another collaborator (Dr. John Brzorad, Reese Institute, Lenoir-Rhyne University) noted: “*I am without words!!! I have been studying these birds since the late 1980’s. And I have never seen them in this way.* [...] *My response is both scientific and emotional. I can see the breeding season, from territory establishment at the colony, brooding, young chicks and large chicks nicely correlated with the number of trips made.*”

As demonstrated, the flexible design of DynamoVis presents the user with a wide selection of various visual elements (points, lines, vectors) for mapping the paths of movement and a range of visual variables (color, transparency, size, texture) for highlighting internal and external attributes along the paths of movement. However, the proper selection and combination of these variables require a systematic user study evaluation to assess their efficacy in enhancing cognition of movement patterns for different applications. For more information on the cartographic design and symbology choices, the readers are referred to Dodge and Noi [1] which provides a systematic classification of available movement visualization techniques and a detailed discussion on various elements of the cartographic framework used in DynamoVis. In addition, our ongoing work focuses on the systematic evaluation of this cartographic framework for various applications. We find, for instance, that animated visualizations of movement have the advantage over static visualizations of allowing for more granular interpretation of movement patterns such as stopping (pausing). Based on comparison of visual variables used to encode movement tracks, we also find line-based visualizations were more effective than dot-based visualizations for the accurate interpretation of overall movement direction along the track. Therefore, further assessment of the cartographic framework presented in Fig. 1 can help inform both the design of DynamoVis and its applications for enhanced visual communication and data exploration.

Although DynamoVis is mainly designed for visualization of animal tracking data, it is equally capable of mapping and animating human tracking data. In this article, we did not describe a use case with human movement due to privacy issues, as the visualizations can easily reveal the location and movement behavior of the tracked individual. However, the software can easily be used with human tracking data for research purposes, if proper precautions are considered for ethical issues. These issues should also be considered in dissemination of visual products created using tracking data of endangered species.

## Conclusion and future developments

This paper introduces DynamoVis 1.0—an open-source visualization software for exploratory analysis and mapping of animal movement tracks and their associations with behavioral and environmental correlates. Proper visualization of these patterns can help inform scientists about how animals adapt to human-induced environmental changes, and help support policy decisions. DynamoVis enables users to quickly and easily create expert-level visualizations based on cartographic theories, with the click of a few buttons, and without any need for programming or expertise in GIS software. DynamoVis empowers movement ecologists to easily display their data through customized interactive visualizations and to animate tracking data sets through the flexible representation of diverse movement context variables. It can be used as decision-support tool to foster discussion and scientific discovery in collaborations between professionals of diverse backgrounds. It also allows users to communicate patterns of movement and disseminate research outcomes by exporting dynamic visualizations in image and video format. Similar visualizations can be created using GIS software or programming approaches. These tools, however, require specialized knowledge of programming or GIS systems, making them difficult for movement ecologists, field biologists, decision-makers, and the general public to use for movement data exploration and knowledge communication. DynamoVis decreases the degree to which a user must understand GIS or data visualization techniques in order to create advanced interactive and dynamic multivariate maps of movement. Additionally, it allows for the easy integration of complex internal and external context information by mapping attributes recorded from modern bio-loggers and global environmental data sets (for example, using the Env-DATA Track Annotation Service) to existing movement data sets. The DynamoVis software is an important research and pedagogical tool for movement ecologists with powerful customization capabilities that require minimal prior data visualization experience and knowledge of cartographic techniques. It is important to note that the visual products of DynamoVis are not intended to replace data analysis and quantitative reasoning, rather they can be used to support decision-making and facilitate analysis through visual reasoning.

In the future release, we plan to implement features for an additional selection functionality in the timeline, enhanced data input capacities for a variety of tracking data formats, display an interactive 3D space-time cube of movement, interaction analysis between multiple moving individuals, and home-range boundary analysis. These new analyses and features are currently under development, and will expand the types of exploratory visual analysis methods available and most beneficial to movement ecologists and other researchers working with movement tracking data. DynamoVis as an open-source software is freely available for the use of scholars and educators to support open science and knowledge communication. We welcome collaborations from the developers and experts from the movement ecology community to enhance the future versions and add specialized visual analytic features.

## Data Availability

The software is freely released under Open Source GNU General Public License 3 on the MOVE Lab’s Github page. Download and install instructions of the source code are available in the repository’s description, and pre-built JAR files are available on the releases page. We welcome contributions to the source code as pull requests and encourage reporting any issues and feedback publicly in the same repository. The Turkey Vulture data are accessible through https://doi.org/10.5441/001/1.46ft1k05 [7], and the Galapagos Albatross are accessible through https://doi.org/10.5441/001/1.3hp3s250 [4]. **Project name:** DynamoVis. **Project home page:** www.github.com/move-ucsb/DynamoVis **Operating system(s):** Platform independent. Software was tested on Windows and MacOS. **Programming language(s):** Java, Processing. **Other requirements:** Java 15 or higher **License:** GNU GPL v3.0. **Any restrictions to use by non-academics:** none. The Galapagos Albatrosses and Turkey Vulture data sets used in this paper are available in the Movebank repository, under a Creative Commons 1.0 Universal (CC0) license.

## Abbreviations

GIS: Geographic Information Systems
GUI: Graphical User Interface
CSV: Comma-separated values
OOP: Object-Oriented Programming
AWT: Abstract Window Toolkit
GPS: Global Positioning Systems
WGS84: World Geodetic System
RAM: Random-access memory
FPS: Frames-per-second
NDVI: Normalized-difference vegetation index
NPP: Net primary production

## Footnotes

1 https://www.movebank.org/cms/movebank-main.

2 https://github.com/move-ucsb/DynamoVis.

3 http://vocab.nerc.ac.uk/collection/MVB/current.

4 Derived variables from ECMWF global atmospheric reanalysis (http://www.ecmwf.int).

5 NASA Land Processes Distributed Active Archive Center, USGS/Earth Resources Observation and Science Center, Sioux Falls, SD, USA.

6 Oregon State Ocean Productivity Data (http://sites.science.oregonstate.edu/ocean.productivity).

7 https://github.com/move-ucsb/DynamoVis.

## Declarations

### Data accessibility

The tracking data used in this paper are available on Movebank (http://www.movebank.org) and are published in the Movebank Data Repository. The Turkey Vulture data are accessible through https://doi.org/10.5441/001/1.46ft1k05 and the Galapagos Albatross are accessible through https://doi.org/10.5441/001/1.3hp3s250.
